# OA19 A complex case of bilateral pneumothoraces due to probable certolizumab driven rheumatoid lung nodules

**DOI:** 10.1093/rap/rkad070.019

**Published:** 2023-09-27

**Authors:** Sajeel Ahmed, Avinash Aujayeb, Ben Thompson

**Affiliations:** Newcastle Hospitals NHS Foundation Trust, Newcastle Upon Tyne, United Kingdom; Northumbria Healthcare NHS Foundation Trust, Cramlington, United Kingdom; Newcastle Hospitals NHS Foundation Trust, Newcastle Upon Tyne, United Kingdom

## Abstract

**Introduction:**

Rheumatoid arthritis (RA) is a chronic, systemic inflammatory disease with articular and extra-articular manifestations. The skin is the most common site of extra-articular involvement and subcutaneous nodules are evident in 25% of RA patients. These are granulomatous nodules often found at extensor surfaces but have also been reported in the menisci, the heart and the lungs. In contrast to rheumatoid nodules, accelerated nodules have a quicker onset and growth and adopt a different dissemination pattern. The case presented here is a patient with seropositive RA who developed accelerated nodulosis likely with the use of certolizumab and consequently required complex intervention.

**Case description:**

A 31-year-old woman, with a background of seropositive (anti-CCP 261 and RF 30), non-erosive rheumatoid arthritis (RA) diagnosed 6 years ago and recent onset of subcutaneous nodules presented with a large right sided spontaneous pneumothorax. She was a never smoker and had no significant family history. She had been on certolizumab 200mg fortnightly via subcutaneous injection for 18 months, hydroxychloroquine 200mg once a day and leflunomide 20mg once a day for 3 years and had trialled methotrexate 3 years previously. With the premise that this was a primary pneumothorax, ambulatory management was initiated with an 8FG Rocket® Pleural Vent™. A repeat CXR showed a small right residual pneumothorax, a new small left pneumothorax and a subtle cavitatory lesion. A chest computed tomogram (CT) showed bilateral small pneumothoraces and cavitating lesions in both lungs. There was no associated lymphadenopathy or pulmonary emboli. The differential diagnoses of such lesions included infection, infarcts, disseminated malignancy and in her case, rheumatoid lung nodules. A chest radiograph done 6 months before the above presentation was normal. Given the rapid appearance of her pulmonary nodules, she was felt to have accelerated nodulosis, potentially secondary to certolizumab or less likely, leflunomide. Due to a persisting air leak, a right-sided video-assisted thoracoscopy, bullectomy, talc pleurodesis and nodule resection was performed. She then presented with a large left sided pneumothorax and similar surgery was also performed. She required prolonged antibiotics and ambulatory drainage due to post-operative empyema. Histology of the cavitatory lesions showed multiple aggregate necrobiotic granulomatous areas surrounded by histiocytes and chronic inflammatory cells. The necrobiotic inflammation extended to the pleural surface and was consistent with the clinical-radiological impression of rheumatoid nodules. Certolizumab and leflunomide were stopped indefinitely and the patient made a complete gradual recover from a pulmonary perspective. She has since been cautiously stabilised on upadacitinib.

**Discussion:**

Given the rapid onset of nodulosis in close relation to introduction of certrolizumab and negative staining for stains such as Ziehl-Neelsen and Grocott, it was deemed that the patient represented a case of accelerated nodulosis. Anti-TNF drugs are recognised to cause reactivation of latent TB and therefore exclusion of infections, such as pulmonary tuberculosis played a significant component of management in this patient. Although, the patient slowly improved from her lung nodulosis and infection perspective after omitting certolizumab and lefluonomide, her RA became more active. However, in view of ongoing empyema requiring long-term chest drain and prolonged antibiotics course, re-introducing immunosuppression with another disease modifying drug represented a difficult treatment strategy. She was, consequently, managed with a slow, tapering course of oral steroids over 20 months with infrequent courses of intra-articular and intra-muscular steroid injections along with hydroxychloroquine. Once fully recovered from infection, she was commenced on 6-monthly 2 pulses of rituximab. As TNF-a level is low in nodules, rituximab is thought of a good treatment option rather than prioritizing anti-TNF drugs in those with accelerating nodulosis. However, our patient had minimal clinical effect from this and after 2 paired pulses was switched to JAK-1 inhibitor upadascitinib along with sulfasalazine and hydroxychloroquine. Her disease has remained quiescent on this regime with no worsening of subcutaneous nodules clinically and repeat CT-thorax a year later revealed no new or residual nodules.

**Key learning points:**

Interstitial lung disease, progressive airway obstruction, nodules, pleural effusions, pneumothorax and pulmonary vascular disease can affect patients with RA. Rheumatoid nodules most commonly occur subcutaneously, and are due to an intense inflammatory process causing fibrinoid necrosis and macrophage activation, leading to an immune-mediated granuloma. Rheumatoid nodules have a prevalence of < 0.4% in radiological studies to 32% in lung biopsies, and are usually commoner in males with positive rheumatoid factor, smokers, and in those with skin nodules and long-term methotrexate. They are usually asymptomatic and do not require intervention. Cavitation may occur and if pleural contact is present, can be associated with a pleural effusion and a pneumothorax. Accelerated nodulosis has been seen with methotrexate, leflunomide, azathioprine, etanercept and drugs such as infliximab and tocilizumab, with rupture of nodules causing pleural disease. The pathogenesis of drug-related nodulosis is not clear. Accelerated pulmonary nodulosis secondary to certolizumab has not been previously described in the literature. Elevated levels of TNF-α are implicated in the pathogenesis of RA and found in the synovial fluid of RA patients and certolizumab is a PEGylated Fab’ fragment of a monoclonal antibody specific to TNF-α and is licensed for use in RA. Certolizumab has shown efficacy with combination methotrexate and as monotherapy in active RA. There is no established treatment for accelerated rheumatoid nodulosis; therefore, it is reasonable to stop the offending drug. With a better understanding of the pathogenesis of accelerated nodulosis, new treatment options may emerge in the future. Thereby, this case highlights the importance of investigating for pulmonary nodulosis in RA patients presenting with new onset respiratory symptoms and altering treatment on a case-by-case basis.

